# C_18_-diterpenoid alkaloids in tribe *Delphineae* (Ranunculaceae): phytochemistry, chemotaxonomy, and bioactivities†

**DOI:** 10.1039/d1ra08132b

**Published:** 2021-12-22

**Authors:** Yuanfeng Yan, Xing Li, Ze Wang, Xiaoyan Yang, Tianpeng Yin

**Affiliations:** Zhuhai Key Laboratory of Fundamental and Applied Research in Traditional Chinese Medicine, Zhuhai Campus of Zunyi Medical University Zhuhai 519041 China ytp@zmu.edu.cn ouyangxiangyan@126.com

## Abstract

This review systematically summarizes the C_18_-diterpenoid alkaloid (DA) compositions isolated from the genera *Aconitum* and *Delphinium* in the *Delphineae* tribe (Ranunculaceae). A total of 117 distinct C_18_-DA components have been reported, including 58 lappaconitine-type DAs, 54 ranaconitine-type DAs, and five rearranged-type DAs. These components mainly originated from plants from the subgenus *Lycoctonum* in the genus *Aconitum* or less frequently from plants within the genus *Delphinium*. Natural C_18_-DAs have exhibited a wide range of bioactivities, including analgesic, antiarrhythmic, anti-inflammatory, anti-tumor, and insecticidal activities, which are closely related to their chemical structures. The high chemical and biological diversities among the reported C_18_-DA constituents in *Delphineae* plants indicated their potential as a vast resource for drug discovery. Additionally, the *Delphineae* plant C_18_-DAs exhibited chemotaxonomic values and showed a high regularity of distribution at different taxonomic levels; therefore, the *Delphineae* plant C_18_-DAs can serve as good chemical molecular markers in the taxonomic treatment of plants within this tribe, especially in the infrageneric division.

## Abstract 2

This review systematically summarizes the C_18_-diterpenoid alkaloid (DA) compositions isolated from the genera *Aconitum* and *Delphinium* in the *Delphineae* tribe (Ranunculaceae).

## Graphical abstract

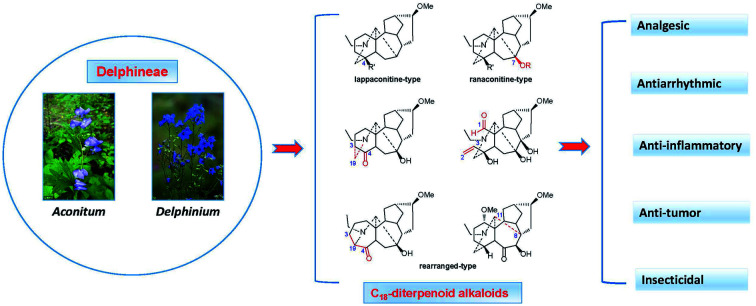

## 1. Introduction

The *Delphineae* tribe is morphologically characterized by zygomorphic flowers in the Ranunculaceae family, which consists of two species-rich genera, *i.e.*, *Aconitum* L. and *Delphinium* L., while the latter also includes the genera *Consolida* Gray, *Aconitella* Spach, and *Staphisagria* J. Hill.^1,2^ This tribe comprises 700–800 species with approximately 350–400 species per genus, which amounts to nearly a quarter of all Ranunculaceae species.^3–5^*Delphineae* plants are distributed mainly in northern temperate regions, including in Asia, Europe, and North America, and occasionally in Africa.^4,6^ The center of diversity and speciation of this tribe is in the eastern Himalayas and southwestern China, as approximately 166 species of *Aconitum* and 150 species of *Delphinium* have been found in this region.^4,5^

Many species within the *Delphineae* tribe are highly valued as medicinal or ornamental plants and have been extensively utilized by various civilizations worldwide since antiquity. In many countries and regions, mainly in the Mediterranean and Asia, various *Delphineae* plants have been extensively employed as herbal medicines for thousands of years to treat multiple kinds of diseases, including rheumatism, traumatic injury, influenza, oedema, enteritis and stomach ache, fainting, various tumors, asthma, skin diseases such as ringworm and scabies, sciatica, migraine, arthralgia, toothache, neuralgia, and other kinds of pain.^7–9^ Especially in China, in addition to two *Aconitum* species officially listed in the Chinese Pharmacopoeia (*A. carmichaelii* Debeaux and *A. kusnezoffii* Reichb.), at least 76 species of *Aconitum* and 32 species of *Delphinium* are used as folk medicines due to their unique and proven therapeutic effects.^10,11^ On the other hand, *Delphineae* plants, especially plants from the genus *Delphinium*, feature various coloured flowers ranging from white, yellow, and red to blue, which have been widely cultivated for centuries as horticultural plants. Currently, some *Delphinium* species, such as *D. elatum* L., *D. grandiflorum* L., *D. ajacis* L. (*C. ajacis* Schur), have become one of the most famous and popular horticultural plants around the world, especially in Europe and America.

*Delphineae* plants have been phytochemically studied since the early 18^th^ century. After hundreds of years of unremitting efforts on exploring their compositions, a large number of metabolites belonging to multiple types of natural products, including diterpenoid alkaloids (DAs), flavonoids, phenic and acids, steroids, and volatile components, have been reported.^8,12^ DAs have been acknowledged as the most characteristic and representative competents for the Delphinieae tribe, as it was reported that nearly 90% of naturally occurring DAs were found in this tribe.^13^ DAs could be further divided into four categories as C_18_-, C_19_-, C_20_-, and bis-types according to the number of carbons in their skeleton. Among them, C_18_-DAs are a highly specific group of compounds. Despite the small amount, they showed great research potential for their novel structures and broad bioactivities: previous studies have revealed a wide range of pharmacological actions for C_18_-DAs, including analgesic, antiarrhythmic, anti-inflammatory, anti-tumor, and insecticidal activities. In particular, the representative C_18_-DA lappaconitine (LA, 37) demonstrated prominent analgesic and antiarrhythmic effects and has been introduced as analgesic (China) and antiarrhythmic (Uzbekistan) drugs since the 1980s. These findings underscore the still large potential of C_18_-DAs in drug discovery and encourage further extensive investigation.

Additionally, the Delphinieae tribe is fame for its taxonomical complexity, as it possesses various complex morphological variations that lack clear relevance, and as a result, the taxonomy of this tribe is very challenging for botanists. Despite its long investigative history involving various systematic methodologies, the taxonomic treatment within this tribe, especially the infrageneric division and the species circumscription, is still frequently discussed and may remain unresolved for many years to come.^14^ Thus, using chemotaxonomy to assist and supplement the investigation is very important. Currently, in addition to zygomorphic flowers, the presence of DAs has been recognized as synapomorphy for this taxonomic group. Moreover, since Ichinohe proposed in 1978 that DAs could reflect the phylogenetic relation of *Delphineae* plants,^15^ DA chemotaxonomic values have been well-illustrated and widely accepted.^16,17^ Applying DAs to address corresponding taxonomic problems has been reported.^18,19^ However, most of these studies were focused on the more widespread DA C_19_- and C_20_-subtypes, and little attention has been given to the less common C_18_-subtypes. C_18_-DAs also possess great chemotaxonomic value, and could serve as a beneficial supplement to conventional systematic taxonomic approaches and provide useful information within this taxonomic phytogroup.

There are several previously published review articles and monographs involving C_18_-DAs,^13,20,21^ but they mainly focused on the progress of studies involving all types of DAs, and only a small portion of research has been devoted to C_18_-DAs. The research by Wang deserves more attention, as the work includes plentiful and varied descriptions of 78 C_18_-DAs with literature coverage to the end of July 2008.^22^ During the past decades, a number of new C_18_-DAs have been reported, and some of them possess previously undescribed C_18_-DA skeletons or impressive bioactivities. Hence, this review was prepared to summarize the research progress on phytochemistry, chemotaxonomy, and bioactivities of natural C_18_-DAs, which will facilitate further research and exploitation of these types of compounds and the utilization of Delphinieae plants.

## 2. Phytochemical studies of C_18_-DAs

Although the first C_18_-DA, LA (37), was isolated in 1895,^23,24^ with its structure confirmed in 1969,^25^ and several representative C_18_-DAs, *e.g.*, lappaconidine (35),^26^ aconosine (4),^27^ and excelsine (29),^28^ were also discovered during the 1970s, the subtype of C_18_-DAs was defined much later. LA and its analogs have been structurally treated as C_19_-DAs for many years. However, in 1983, Wang *et al.* suggested to use C_18_-DAs for these alkaloids to distinguish them from C_19_-DAs,^29^ as they possess structural features that are distinctive compared to those of C_19_-DAs, namely, C_18_-DAs lack C-18, which generally appears as methyl or oxygenated methylene in C_19_-DAs. As the quantity of C_18_-DAs increased over decades, the term C_18_-DAs was gradually accepted. Currently, C_18_-DAs are mostly regarded as an independent group within DAs, which are also called “bisnorditerpenoid alkaloids”.^20^

**Fig. 1:**
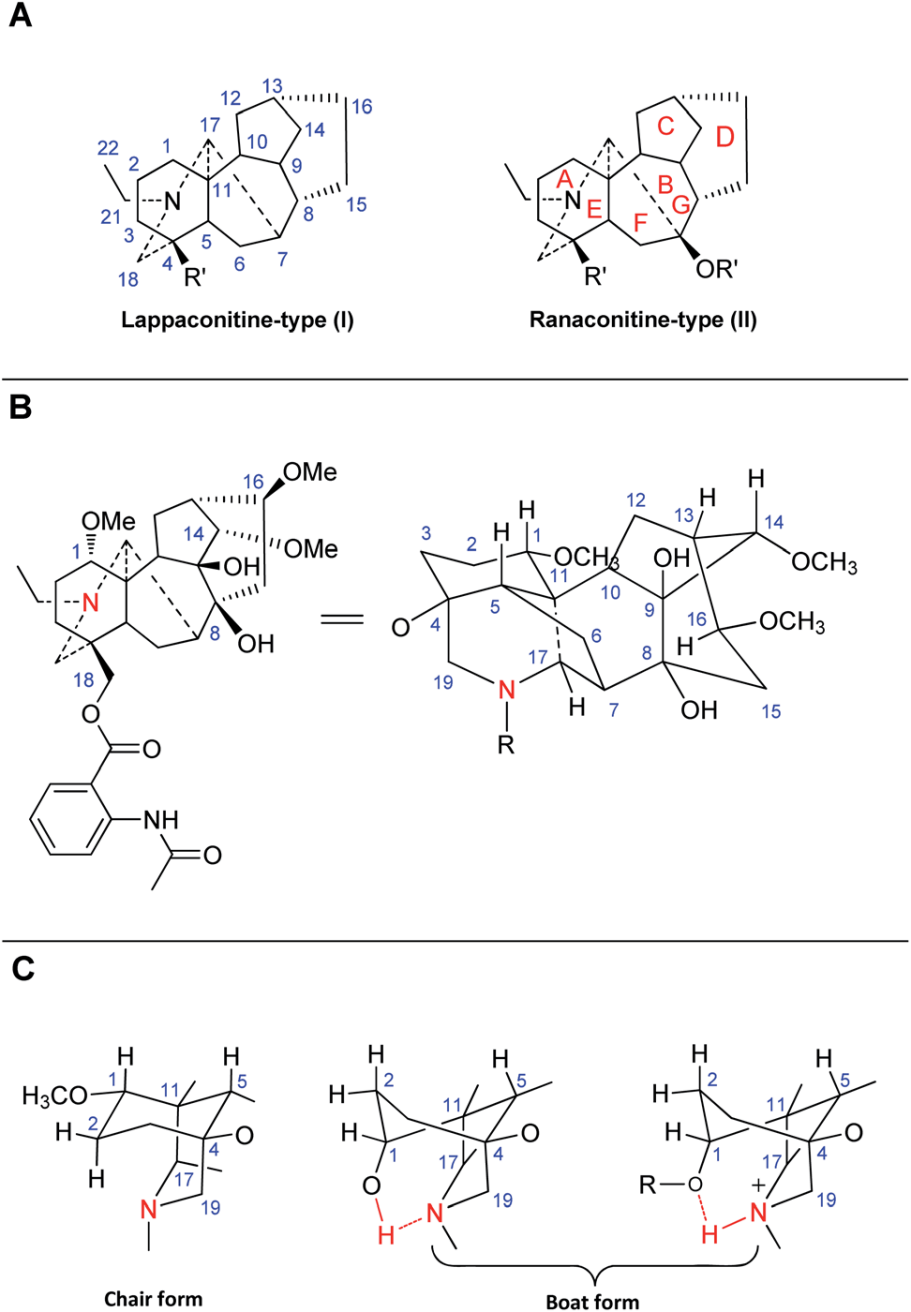
(A) Two typical subtypes of C_18_-DAs; (B) the structure of LA (projection formula); (C) the conformations of ring A in C_18_-DAs.

C_18_-DAs are usually classified into two typical subtypes based on whether an oxygen-containing functionality (*e.g.*, OH, OMe, or OCH_2_O) is attached at C-7, namely, lappaconitine-type DAs (I), which do not possess an oxygen-containing functionality at C-7, and ranaconitine-type compounds (II), which do have an oxygen-containing functionality at this position (Fig. 1A).^22^ C_18_-DAs belonging to these two subtypes possess a heptacyclic framework, comprising four six-membered rings (A, B, D and E), including one six-membered N containing heterocyclic ring (E), and two five-membered rings (C and F). According to single crystal X-ray diffraction analysis of corresponding C_18_-DAs, *e.g.*, LA (37, Fig. 1B), sepaconitine (46),^30^*N*-deacetyllappaconitine (41),^31^ and ranaconitine (102),^32^ the C_18_-DAs possess stable conformations for most of the rings except for rings A and D, as they only have one or two flexible atoms in the skeleta, which is identical to that of C_19_-DAs.^33,34^ Generally, rings C and F in C_18_-DAs exist in envelope form, rings E and G adopt chair form, and ring B is fixed in a boat conformation. The conformation of ring A in C_18_-DAs (as well as in C_19_-DAs) is mainly affected by the substituents at C-1 and the protonated N. These C_18_-DAs with OMe-1 or OAc-1 possess a chair conformation of ring A, while C_18_-DAs with OH-1 substituent have a ring A in boat conformation, which is stabilized by an intramolecular hydrogen bond between OH-1 and the lone electron pair at N (Fig. 1C).^35,36^ In addition, protonation of N also resulted in a boat conformation of ring A due to the intramolecular hydrogen.^37^ The conformation of ring D in C_18_-DAs is more complicated, and is associated with the substituents at C-13, C-15, and C-7. The fusion of several main rings is identical for all C_18_-DAs: A/B and E/F, *trans*; A/E and B/C, *cis*.

In addition to lappaconitine- and ranaconitine-type C_18_-DAs, several compounds with unprecedented rearranged-type C_18_-DA skeletons (III) have also been discovered in recent years. To date, a total of 117 C_18_-DAs have been reported, and their trivial names, plant origins and references are listed in Table S1.† Herein, phytochemical studies of C_18_-DAs are summarized by category.

### 2.1. Lappaconitine-type

**Fig. 2:**
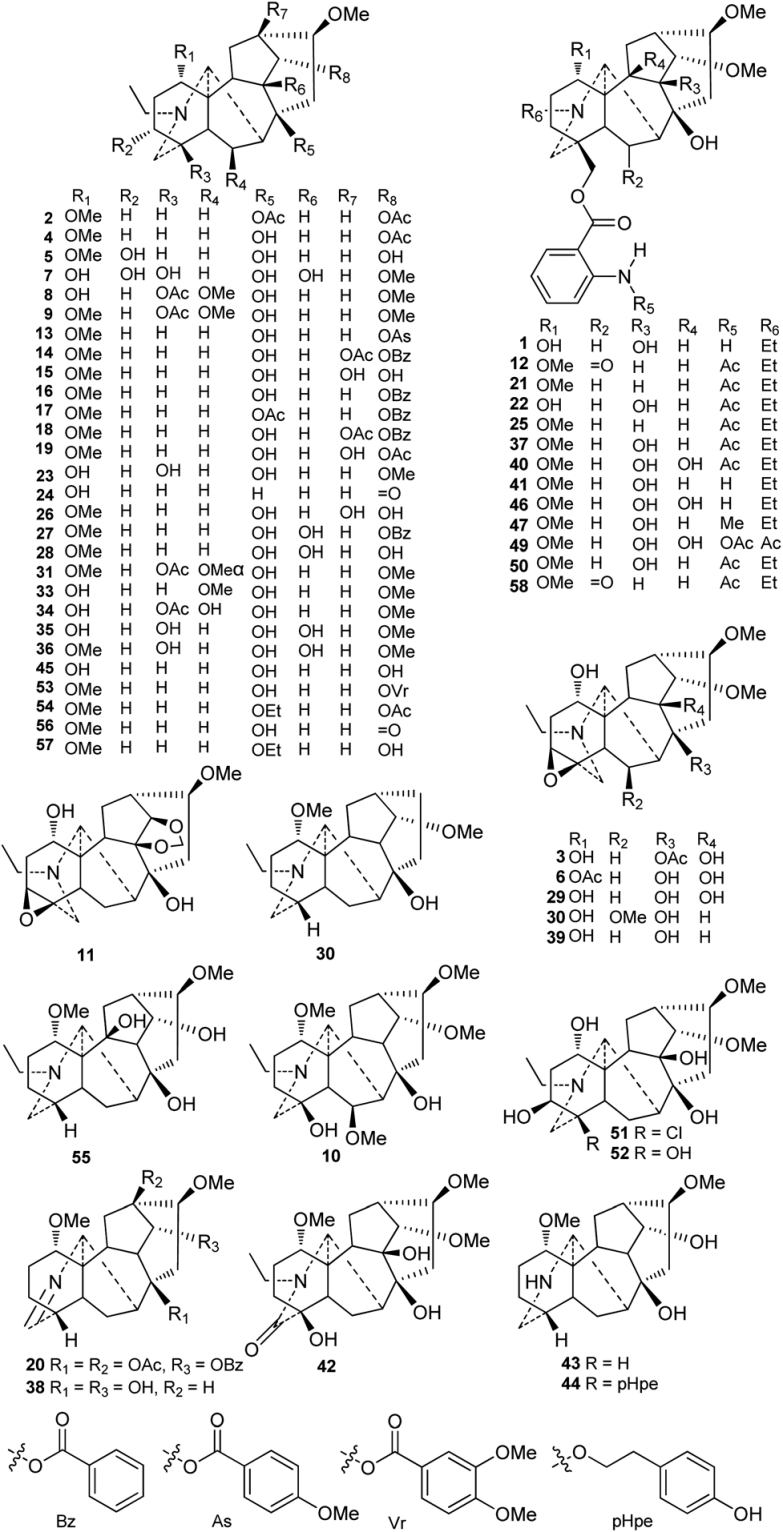
Lappaconitine-type C_18_-DAs (1–58).

Currently, approximately 58 lappaconitine-type C_18_-DAs (type I) have been reported (Fig. 2). Most lappaconitine-type C_18_-DAs were found in the *Aconitum* species, while only a few exceptions have been reported, *e.g.*, giraldine I (30) from *D. giraldii* Diels^38^ and 6-ketoartekorine (58) and artekorine (12) from *Artemisia korshinskyi* Krash. ex Poljakov in the family Compositae.^39^ In addition, four lappaconitines were found in both *Aconitum* and *Delphinium* plants, namely, delphicrispuline (21),^40,41^ lappaconidine (35),^26,42,43^ puberanidine (41),^44–46^ and sinomontanine A (50).^47,48^ Most of the lappaconitine-type C_18_-DAs are scattered in certain *Aconitum* species with a narrow distribution, and only a few of them have a relatively wide distribution, such as aconosine (4), dolaconine (26), lappaconidine (35), and puberanidine (41).

The structural diversity of C_18_-DAs is mainly determined by the state of the N atom and the oxygenated substituents that vary in their variety, quantity, position, and orientation. The N atom usually presents as a tertiary amine (NR_3_) with a diagnostic *N*-ethyl group. Among the reported lappaconitines, piepunendines A and B (43 and 44) from *A. piepunense* Hand.-Mazz. are characterized by the absence of the typical *N*-ethyl group,^49^ resulting in a secondary amine (NHR_2_), and sinaconitine B (49) from *A. sinomontanum* Nakai features a rare N–C_(21)_=OMe acetamide group instead of the *N*-ethyl group.^50^ In addition, 19-oxolappaconine (42) from *A. septentrionale* Koelle possesses an N–C_(19)_=O lactam group,^51^ which might be formed by the oxidization of OH-19. Delavaconitine G (20) from *A. delavayi* Franch.^52^ and liconosine A (38) from *A. forrestii* Stapf^53^ possess an uncommon N=C_(19)_ imine group.

Similar to C_19_-DAs, hydroxyl (OH) and methoxyl (OMe) are the most common oxygenated substituents in lappaconitines. The methoxyl groups are mainly located at C-1, C-6, C-14, and C-16. Almost all of the reported lappaconitines contain OMe-16β with the exception of giraldine I (30), which lacks an oxygen-containing functionality at this position.^38^ In this type of DA, methoxyl groups at C-1 and C-14 are fixed in the α-orientation, while OMe-6 might have either an α- (*e.g.*, compounds 31 and 32) or β-orientation (*e.g.*, compounds 8–10). The OH groups are mainly distributed at C-1, C-4, C-8, and C-14 and are occasionally distributed at C-3, C-6, C-9, C-10, and C-13, and are easily esterified by various ester groups, including acetyl (Ac) and aroyl groups such as benzoyl (Bz), anisoyl (As), veratroyl (Vr), or anthranoyl.^54^ These aroyl groups have preferred substituent locations; for example, the Bz, As, and Vr groups are always substituted at C-14, and the anthranoyl group is exclusively located at C-18.

Six alkaloids featuring a 3,4-epoxy group were reported, including 8-acetylexcelsine (3),^55^ akirine (11),^56^ excelsine (29),^28^ kiritine (32),^57^ monticamine (39),^58^ and akiranine (10),^59^ which could be regarded as the characteristic substituents of C_18_-DAs that distinguish them from C_19_-DAs. There are two alkaloids, weisaconitines A and D (54 and 57), from *A. weixiense* W. T. Wang, which rarely have an oxyethyl at C-8.^60^ In addition, several lappaconitines that possess uncommon substituents have been reported; for example, sinomontanine N (51) from *A. sinomontanum*^61^ contains a chlorine (Cl) at C-4, which is rare in secondary metabolites produced by terrestrial plants, and piepunendine B (44) from *A. piepunense* has a unique 2-(*p*-hydroxyphenyl)ethoxy group at C-8.^49^

### 2.2. Ranaconitine-type

**Fig. 3:**
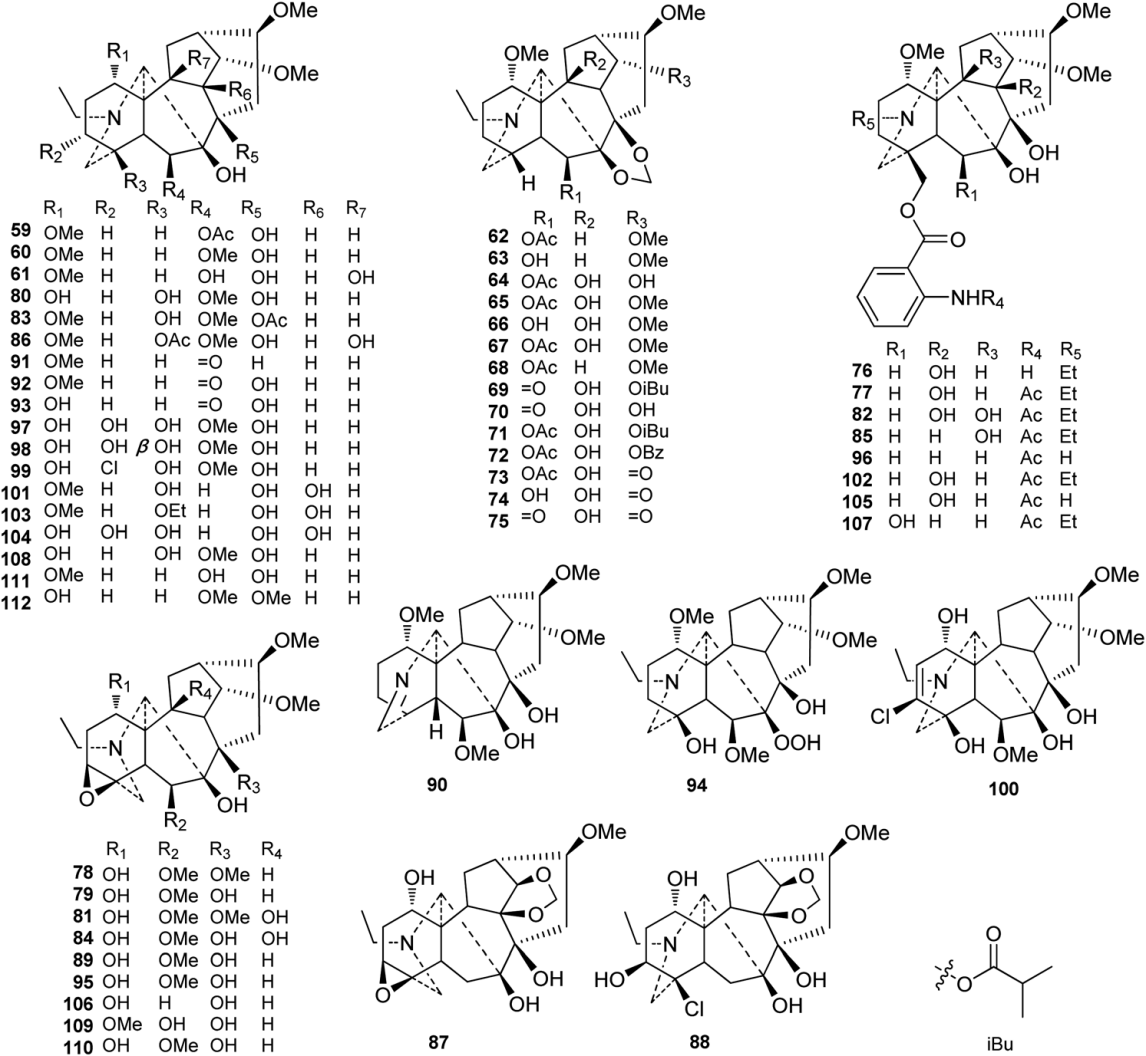
Ranaconitine-type C_18_-DAs (59–112).

To our knowledge, 54 ranaconitine-type C_18_-DAs (type II) were reported from *Delphineae* plants (Fig. 3). Similarly, most of the ranaconitine-type C_18_-DAs were found in *Aconitum* plants. However, a certain number of ranaconitines are distributed in *Delphinium* plants. Most ranaconitines are tertiary amines, which is consistent with lappaconitine-type DAs, and only a few expectations have been reported, namely, imine lamarckinine (90)^62^ and secondary amines puberanine (96)^44^ and sinomontanine H (107).^63^ Although the variety of substituents in ranaconitines and lappaconitines is roughly identical, ranaconitines possess more oxygenated substituents. In addition to their oxygenated substituent at C-7, ranaconitines are also easier to be substituted by =O, or OH, OAc, and OMe with a β-orientation at C-6. The dioxymethylene group (OCH_2_O) is more frequent in this type of compound and is mainly located at C-7/C-8. In rare cases, two alkaloids bear an OCH_2_O group between C-10 and C-14, namely, kirisines A and B (87 and 88) from *A. barbatum* Pers. (synonym *A. kirinense*).^64^ A series of ranaconitines possessing OH-10, including anthriscifolcines C-G (65–68), anthriscifolcones A and B (69 and 70), and anthriscifoltines C-G (71–75), were found in two varieties of *D. anthriscifolium* Hance.^65–68^ Among them, anthriscifolcines F and G (67 and 68) also feature an OH-16 substituent instead of the common OMe-16 substituent. There are also a series of ranaconitines bearing the characteristic 3,4-epoxy group that have been reported.^69–72^ Several ranaconitine-type compounds that feature rare substituents have also been reported; for example, puberumines C and D (99 and 100) from *A. barbatum* var. *puberulum* Ledeb. Fl. Ross. that possesses a chlorine at C-3 was reported.^58^ In addition, lineariline (94) from *D. linearilobum* (Trautv.) N. Busch contains a rare peroxyl group (OOH) at C-7.^73^

### 2.3. Rearranged-type

**Fig. 4:**
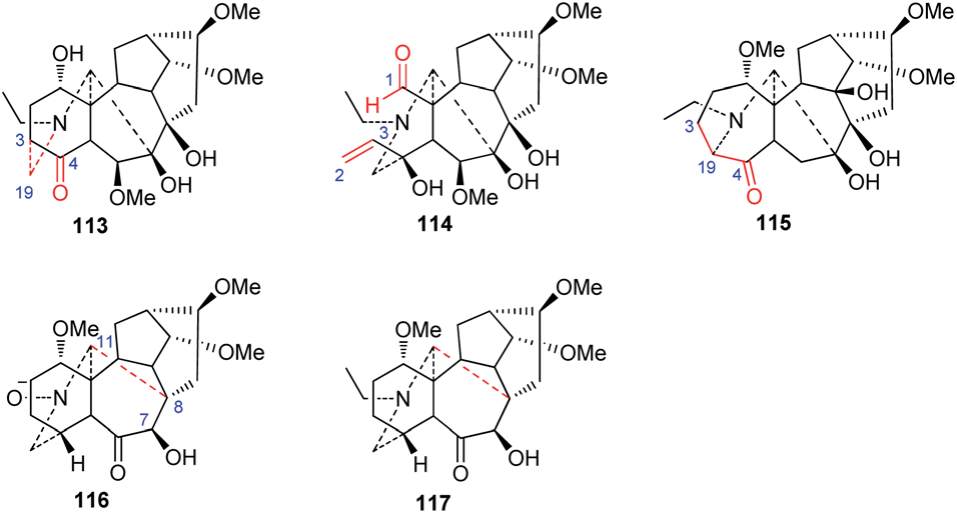
Rearranged-type C_18_-DAs (113–117).

New C_18_-DA subtypes have seldom been reported—only four rearranged C_18_-DA skeletons have been discovered in recent decades (Fig. 4). Among them, puberudine (113) and puberunine (114) are two novel C_18_-DAs isolated from *A. barbatum* var. *puberulum*.^58^ The former alkaloid (113) possesses a novel seven-membered E ring, in which the typical C_(19)_–C_(4)_ bond is rearranged to form a new C_(19)_–C_(3)_ bond, and the latter (114) contains a seco A ring that is generated *via* C_(1)_–C_(3)_ bond cleavage, forming an extra CHO-1 group and a Δ^2,3^ unit in the A ring. Sinomontadine (117) from *A. sinomontanum* features a rearranged seven-membered A ring, which might be formed by the incorporation of C-19 into the normal C_(3)_–C_(4)_ bond.^61^ It is worth noting that the structure of sinomontadine (117) was confirmed by single-crystal X-ray diffraction experiments. Two C_18_-DAs with an unusual E ring similar to acoseptine-type C_19_-DA were also reported,^74^ namely, barpubenines A and B (114 and 115) from *A. barbatum* var*. puberulum*,^75^ in which the C_(7)_–C_(17)_ bond was rearranged to a C_(8)_–C_(17)_ bond, forming an additional ketone at C-7. In addition, barpubenine A is the only *N*-oxide in C_18_-DAs. In summary, these alkaloids represent the new subtypes of C_18_-DAs.

## 3. Chemotaxonomy

DAs have been acknowledged as good chemical molecular markers in the chemotaxonomy of *Delphineae* plants and have played a vital role in the taxonomy of the *Delphineae* tribe, especially the C_19_ and C_20_ subtypes of DAs, which have extensively clarified chemotaxonomic values and have been applied in many cases.^18,76,77^ However, in contrast to the unremitting efforts in exploring C_18_-DA components with novel structures in *Delphineae* plants, much less attention has been given to their chemotaxonomic values. The potential role that C_18_-DAs might play as chemical markers in the taxonomy of *Delphineae* plants has been largely ignored. Hence, the chemotaxonomic value of C_18_-DAs in the *Delphineae* tribe is discussed herein to facilitate the knowledge of Delphinieae plant taxonomy.

**Fig. 5:**
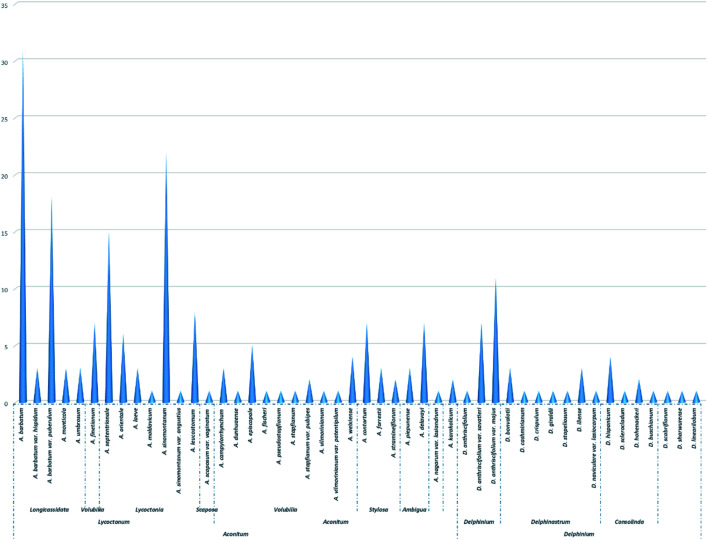
The distribution of C_18_-DAs in tribe *Delphineae*.

As shown in Fig. 5 and Table S2,† C_18_-DAs are mainly distributed in *Aconitum* plants—nearly 80% of C_18_-DAs for types I, II, and III have been found in *Aconitum* plants. Taxonomically, the genus *Aconitum* is usually divided into three distinct subgenera, *i.e.*, *Aconitum*, *Lycoctonum*, and *Gymnaconitum*, based mainly on their morphological root differences.^4^ Most of the C_18_-DAs were found in plants belonging to the subgen. *Lycoctonum*, while much fewer C_18_-DAs were found in the subgen. *Aconitum*, and none were found in the subgen. *Gymnaconitum*, which contains only one species, *i.e.*, *A. gymnandrum* Maxim. In the approximately 40 species in subgen. *Lycoctonum* worldwide, nearly 13 species or varieties have been found to contain C_18_-DA compositions. C_18_-DAs can be regarded as the predominant chemical constituents of subgen. *Lycoctonum*, which is distinguished from the subgen. *Aconitum* by the large number of aconitine-type C_19_-DAs and much fewer C_18_-DAs.^7^ From the perspective of chemotaxonomy,^16,17^ the evolution degree of DAs increases in the order of C_20_- < C_19_- < C_18_-DAs in terms of structural types, as C_19_-DAs possess more complex polycyclic structures derived biogenetically from C_20_-DAs, while C_18_-DAs are generated by the oxidative degradation of C_19_-DAs. In general, the more C_20_-DAs are distributed in plants, the more primitive the phytogroup; in contrast, the more C_19_- or C_18_-DAs are distributed in plants, the more evolved the phytogroup. Subgen. *Lycoctonum* has been accepted as a primitive group relative to the subgen. *Aconitum*. The fact that this subgenus is abundant in C_18_-DAs could be due to the parallel evolution that exists widely within the *Delphineae* tribe.^16,78^

In addition, within the subgen. *Lycoctonum*, C_18_-DAs are rarely found in primordial species, such as *A. apetalum* (Huth) B. Fedtsch.^79–81^ and *A. brevicalcaratum* Diels^82–84^ in series Crassiflora, which has been certified by extensive phytochemical studies. Conversely, most of the C_18_-DAs are found in species belonging to ser. Longicassidata and Lycoctonia, which represent a relatively evolved position within this subgenus. In particular, several species have been reported to be abundant in C_18_-DAs, including *A. barbatum* (*A. kirinense*), *A. barbatum* var. *puberulum*, *A. sinomontanum*, and *A. septentrionale*. Thus, it can easily be concluded that within the subgen. *Lycoctonum*, an advanced species can possess more C_18_-DAs with abundant structural diversity. Thus, C_18_-DA can serve as an important indicator to reflect the degree of evolution of certain species within this subgenus.

According to collected data, C_18_-DA compositions could also be used to estimate genetic relationships or distinguish sibling species; for example, the occurrence of several C_18_-DAs (35, 36, 37, 41, 46, and 77) in both *A. septentrionale* and *A. sinomontanum* supports the morphological similarities of these taxa, and their apparent differences that may be useful to distinguish these taxa. It is worth noting that these conclusions should not be drawn until extensive phytochemical studies have been performed on the corresponding species.

Almost all of the C_18_-DAs found in subgen. *Aconitum* are lappaconitine-type DAs (Table S3†), and they are distributed mainly in plants belonging to the ser. Volubilia, followed by ser. Stylosa and Ambigua. There are ten species or varieties within ser. Volubilia that have been found to contain C_18_-DAs. It is commonly accepted that ser. Volubilia represents a relative evolutionary status in the subgen. *Aconitum* and is also considered an intermediate transitional phytogroup connecting the ser. Stylosa/Ambigua and ser. Inflata. The distributions of lappaconitine-type C_18_-DAs in these plants clearly revealed the close affiliation between these series within this subgenus. In addition, C_18_-DAs are rarely found in plants from these relatively primordial plants in the subgen, such as the *A. tanguticum* Stapf in ser. Tangutica,^85–87^ and *A. rotundifolium* Kar. et Kir. in ser. Rotundifolia.^88,89^ The component distribution was consistent with the degree of plant evolution within this phytogroup, which demonstrated the chemotaxonomic values of C_18_-DAs as chemotaxonomic markers.

When compared with that of *Aconitum*, much fewer C_18_-DAs have been found in the *Delphinium* genus, and most of the reported C_18_-DAs from *Delphinium* are ranaconitine-type compounds (Table S4†), which can be distinguished from the genus *Aconitum* by the existence of C_18_-DAs that belong to both types I, II, and III. It is also noted that a series of ranaconitine-type DAs were found in two varieties of *D. anthriscifolium*, namely, *D. anthriscifolium* var. *savatieri* and *D. anthriscifolium* var. *majus*,^65–68^ which belongs to the subgen. *Delphinium*. According to the species division of the genus *Delphinium* by Wang,^5^ the subgenus *Delphinium* contains only two species, namely, *D. anthriscifolium* and *D. ludingense* W. T. Wang, while another subgenus, *Delphinastrum*, comprises *ca*. 165 species. The abundance of C_18_-DAs in *D. anthriscifolium* distinguishes it from other *Delphinium* species, which supports the unique taxonomic position of this genus that was obtained by classical taxonomic methodologies. In addition, the occurrence of C_18_-DAs in *Delphinium* also supports the viewpoint that parallel evolution widely exists in plants within this tribe.

In addition, two C_18_-DAs (12 and 58) have been isolated from *Artemisia korshinskyi* in the family Compositae.^39^ However, the data are not sufficient enough to be useful in exploring their chemotaxonomic values.

In summary, C_18_-DAs exhibited some important distribution regularity within tribe *Delphineae*, which occurred as follows: (1) at the genus level, C_18_-DAs are distributed mainly in plants from the *Aconitum* genus and less so from *Delphinium* plants; (2) in terms of the subgenus, the subgen. *Lycoctonum* in the genus *Aconitum* is the richest source of C_18_-DAs, as a large number of compounds belonging to all types of C_18_-DAs have been found in this phytogroup. In contrast, the subgen. *Aconitum* possesses only lappaconitine-type C_18_-DAs in relatively low amounts. The genus *Delphinium* contains mainly ranaconitine-type C_18_-DAs and its subgen. *Delphinium* contributed much more C_18_-DAs than the subgen. *Delphinastrum*; (3) within a certain subgenus, C_18_-DAs are mainly distributed in relatively evolved phytogroups, such as ser. Longicassidata in subgen. *Lycoctonum*, and ser. Volubilia in subgen. *Aconitum.* Sibling spices with close genetic relationships might share some C_18_-DAs. Overall, these findings demonstrated the chemotaxonomic values of C_18_-DAs and support the potential of C_18_-DAs to serve as chemical molecular markers in the taxonomic treatment of plants from this tribe.

## 4. Bioactivities

### 4.1. Analgesic activity

*Delphineae* plants have long been employed for treatment of various kinds of pains in traditional medicines, which could be attributed to their characteristic DA compositions. The analgesic activities of DAs have been investigated since the 1980s, which have led to the development of three DAs as analgesic drugs, *i.e.*, the C_19_-DAs 3-acetylaconitine and crassicauline A and the typical C_18_-DA LA (37). The hydrobromide salt of LA is used in commercial lappaconitine tablets and injection as the first non-addictive analgesic drug in China, and it is extensively employed for the clinical treatment of various types of mild or moderate pain, such as cancer pain, postoperative pain, and sciatica. Compared to these two C_19_-DA analgesic drugs (crassicauline A and 3-acetylaconitine), LA exhibited stronger antinociceptive efficacy and less toxicity. It has been observed in clinical practice that the analgesic effect of lappaconitine are generally approximately seven times greater than that of phenazone, a commonly used non-steroidal anti-inflammatory drug, and is almost equipotent to pethidine, with a longer maintenance time of approximately 2–22 h. Moreover, lappaconitine hydrobromide is non-narcotic, inducing neither morphine-like tolerance nor physical dependence. However, the analgesic effect of lappaconitine is slower than that of pethidine, and its clinical dosage range is relatively narrow; therefore, LA is not suitable for the treatment of severe pain.

In animal experiments, lappaconitine demonstrated unambiguous analgesic effects in various models, including writhing, tail-pinch, hot plate tests in mice, and electric stimulation tests in rats. It is generally accepted that the mechanism at supraspinal levels is responsible for LA analgesia, as it could block voltage-dependent Na^+^ channels in the central nervous system,^90^ affect calcium influx in the midbrain periaqueductal gray (PAG) and the cortex,^91^ stimulate descending pathways related to noradrenalin and serotonin,^92^ inhibit activities of hippocampal neurons,^93^ and decrease the transmission of nociceptive information *via* brain, substance P (SP) and/or somatostatin pathways.^94^ Another study indicated that the analgesic effect of LA might also be involved in the decrease in the expression and sensitization of P2X3 receptors in rat DRG neurons following chronic constriction injury.^95^

**Fig. 6:**
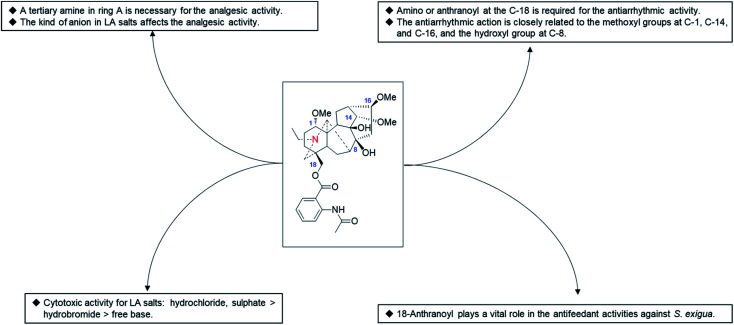
The SAR of LA analogues.

In addition to LA (37), several other natural C_18_-DAs, such as *N*-deacetyllappaconitine^90^ and 8-*O*-ethylaconosine,^60^ also exhibited certain analgesic activity, but little attention has been given to these alkaloids. A SAR study performed by Wang *et al.* revealed that the analgesic activity of LA analogues is closely related to the state of N in ring A and that a tertiary amine is an important structural feature necessary for the analgesic activity of the LA analogues (Fig. 6).^96^ In addition, the analgesic activity of LA salts is also affected by the kinds of ions, for example it was reported that the analgesic effect of LA hydrochloride is worse than that of its hydrobromide,^97^ while another study revealed that LA sulfate exhibited a more pronounced analgesic activity than other salt forms.^98^

### 4.2. Antiarrhythmic activity

Since 1977, when Dzhakhangirov *et al.* first discovered the C_20_-DA napelline and the C_19_-DA heteratisine bearing considerable antiarrhythmic activity, the antiarrhythmic activity of various types of DAs has attracted much interest from scientists. In a large-scale screening for antiarrhythmic compounds from hundreds of DAs performed by Dzhakhangirov *et al.* using the models of aconitine-introduced arrhythmia in anesthetized rats and of irreversible cardiac fibrillation in alert mice,^99^ the representative C_18_-DA LA (37) and its major metabolite *N*-deacetyllappaconitine (41) exhibited pronounced antiarrhythmic and antifibrillatory action, with AAI (LD_50_/ED_50_) values of 118 and 146, respectively, which were more than 1000 times superior in antiarrhythmic activity and more than 50-fold superior in breadth of therapeutic action to the positive control novokainamid. Unlike novokainamid, these DAs also exerted powerful protective antifibrillatory action and prevented the death of animals poisoned with a lethal dose of aconitine, which highlights their potential as antiarrhythmic drugs. Subsequent studies demonstrated the negative inotropic effects of LA (37), which could increase the excitation threshold and eventually cause bradycardia and asystolia.^100^ This typical class-IC antiarrhythmic activity of LA (37) might be due to its electrophysiological properties on sodium channels, which could suppress the fast-incoming sodium current by long-term binding to the site 2 receptor, subsequently decreasing the depolarization rate, leading to a slowing of impulse propagation and a decrease of excitability in the conductive system of the heart.^101^ LA (37) also showed electrophysiological action on other ion currents, such as calcium and potassium, by modulating the expression of related genes, which might also be responsible for its antiarrhythmic activity.^102^ Finally, LA hydrobromide was introduced into medical practice as an antiarrhythmic agent under the name of allapinin in Uzbekistan, which was proven to be effective in the treatment of life-threatening forms of arrhythmias, namely, atrial fibrillation (AF) and ventricle rhythm disorders.^103,104^ Currently, this drug is included in the list of the most vitally important medicines approved by the Ministry of Health of the Russian Federation.

In addition to LA (37) and *N*-deacetyllappaconitine (41), other C_18_-DAs also showed antiarrhythmic activity, and several even exerted a superior antiarrhythmic action to LA (37) in some respects. For example, *N*-acetylsepaconitine (40) and ranaconitine (102) exhibited a stronger antiarrhythmic action in aconitine-introduced arrhythmia in rats, with AAI values of 214 and 124, respectively, which were greater than that of LA. Consistent results were obtained in a recent study which showed that *N*-acetylsepaconitine (40) could significantly prolong the ventricular premature (VP) action in aconitine-induced arrhythmia mice, with an effect better than that of lappaconitine, while their inhibition of ventricular tachycardia (VT) and ventricular flutter (VFL) was similar.^75^ From the data available, the presence of amino or anthranoyl groups at the C-18 position is required for the antiarrhythmic activity of C_18_-DAs, and their antiarrhythmic action is closely related to the methoxyl groups at C-1, C-14, and C-16, along with the hydroxyl group at C-8. Therefore, more efficient antiarrhythmic agents using available natural C_18_-DAs as lead compounds could be designed based on SAR analysis.

### 4.3. Anti-inflammatory activity

Traditionally, *Aconitum* and *Delphinium* plants have been widely used for the treatment of arthritis, which implies that their major constituents DAs possess certain anti-inflammatory activity. Although most of the newly discovered anti-inflammatory compounds from *Delphineae* plants are C_19_-DAs,^105^ there are also several reports that involve C_18_-DAs. For example, ranaconine-type C_18_-DA sinomontanine I (108) from *A. sinomontanum* showed a dose-dependent inhibitory effect on both lipopolysaccharide (LPS) and concanavalin A (ConA) induced splenic lymphocyte proliferation, with IC_50_ values of 8.909 and 3.661 μM, respectively.^106^ Four lappaconine-type C_18_-DAs from *A. fischeri* var. *arcuatu*m (4, 9, 24, and 53) showed weak inhibitory activity against NO production in LPS-induced RAW 264.7 macrophages with an inhibition rate of approximately 30% at a concentration of 40 μM.^107^ In animal experiments, lappaconitine (8 and 16 mg kg^−1^, ig) effectively inhibited edema of the hind paw induced by injection of carrageenin, formaldehyde, and Freund's complete adjuvant (FCA) in rats, restrained swelling of ear induced by xylene in mice, and inhibited the proliferation of granulomas induced by injection of agar in rats.^108^ Another study also reported the anti-inflammatory effect of lappaconitine (37), which could inhibit the edema of the hind paw of rats induced by FCA and reduce the contents of TNF-α, IL-2, CIC and PGEa in their serum.^109^ In general, these studies preliminarily revealed the anti-inflammatory effects of C_18_-DAs *in vitro* and *in vivo*.

### 4.4. Anti-tumor activity

While most of the naturally occurring C_18_-DAs, including lappaconitine (37), which usually presented as freebase, showed only slightly active against human cancer cell lines during the primary screen,^58,110^ a certain degree of anti-cancer effect has also been discovered for several kinds of lappaconitine salts. It was reported that the hydrobromide salt of lappaconitine could suppress the growth of liver and S180 tumors of mice with inhibition rates of 11.20%∼53.08% and 29.81%∼53.96%, respectively^111,112^ and could inhibit the proliferation of human non-small cell lung cancer A549 cells dose dependently by arresting the cells in G1/G0 phase and downregulating the expression of Cyclin E1 (Table 1).^113^ Lappaconitine sulfate was also reported to possess antiproliferative activity against various human cancer cell lines: cerical neoplasm (HeLa),^114^ liver (HepG2),^115^ colon (HT-29),^116^ and lung (A549),^117^ which might be caused by activation of p38 MAPK-, mitochondrial-, and caspase-mediated apoptosis. In addition, the hydrochloride salt of lappaconitine has been observed for its ability to inhibit proliferation and to induce apoptosis in human colon cancer HCT-116 cells and human liver cancer HepG2 cells *via* mitochondrial and MAPK pathways.^118,119^ Generally, the sulfate and hydrochloride salts of lappaconitine exhibited a relatively higher cytotoxic effect than its hydrobromide salt, in combination with the fact that these two salts also possess better water solubility,^98^ indicating their higher potential in tumor therapy.

**Table 1:** Cytotoxic effect of lappaconitine salts (IC_50_, mg mL^−1^, 48 h)

Lappaconitine salts	A549	HeLa	HepG2	HCT-116
Hydrobromide	3.925	—	—	—
Sulfate	0.551	0.421	0.360	—
Hydrochloride	—	—	0.372	0.174

### 4.5. Insecticidal activity

Some species in the tribe *Delphineae* that are rich in C_18_-DA compositions have long been utilized as natural insecticides;^4,73^ therefore, C_18_-DAs might possess insecticidal activities, which has been preliminarily confirmed by several studies. Ulubelen *et al.* evaluated the repellent activity against the warehouse pest *Tribolium casteneum* (Herbst.) of 29 natural DAs isolated from Turkish *Delphineae* plants, including two common C_18_-DAs lappaconitine (37) and *N*-deacetyl lappaconitine (41).^120^ As a result, *N*-deacetyl lappaconitine (41) exerted a relatively high repellency class III effect (40.1–60%) with a repellency value of 50.00% at 3 mg mL^−1^, suggesting that it might be a promising candidate for insecticide development. However, lappaconitine (37) showed only a low-class II repellent effect (20.1–40%), with a repellency value of 34.37% at 3 mg mL^−1^.

In addition, three ranaconine-type C_18_-DAs (110, 79, 78) featuring a 3,4-epoxy group were screened for their insect antifeedant activity against the pests Colorado potato beetle (*Leptinotarsa decemlineata*) and *Spodoptera littoralis*.^121^ These three ranaconines showed a roughly similar effect against *L. decemlineata* with EC_50_ values lower than 5 μg cm^−2^ (Table 2). Alkaloids 110 and 79 also showed certain antifeedant activity against *S. littoralis* with EC_50_ values of 5.38 and 11.79 μg cm^−2^, respectively, while alkaloid 78 was completely invalid against this kind of pest. More recently, another study performed by Chen *et al.* revealed that C_18_-DA components in the Chinese *Aconitum* species *A. leucostomum* possess antifeedant activity against the larvae of *Spodoptera exigua*.^122^ Among the seven tested C_18_-DAs, *N*-acetylsepaconitine (40), *N*-deacetyl lappaconitine (41), and finaconitine (82) with an anthranoyl group at C-18 exerted strong antifeedant activities against *S. exigua* with EC_50_ values lower than 2 μg cm^−2^, followed by acosepticine (111), lappaconidine (35), and leucostonine (93) (EC_50_, 8–21 μg cm^−2^), while leucostine (92) was basically ineffective (EC_50_ > 30 μg cm^−2^). These results revealed that the 18-anthranoyl substituent plays a vital role in the antifeedant activities against *S. exigua* of C_18_-DAs, but more structure–activity relationship (SAR) research is required to confirm this.

**Table 2:** Antifeedant effects of C_18_-DAs (EC_50_, μg cm^−2^, and 95% confidence limits)

C_18_-DAs	*L. decemlineata*	*S. littoralis*	*S. exigua*
78	1.92 (0.66, 5.54)	≈ 50	—
79	2.36 (0.47, 11.80)	5.38 (1.43, 20.37)	—
110	3.31 (1.10, 9.94)	11.79 (11.70, 11.89)	—
35	—	—	17.65 (11.10, 28.07)
40	—	—	<2 (0.85, 1.73)
41	—	—	1.88 (1.12, 3.18)
82	—	—	1.45 (0.75, 2.81)
92	—	—	>30
93	—	—	20.75 (14.09, 30.54)
111	—	—	8.59 (5.98, 12.36)

## 5. Conclusions

This review systematically summarizes the C_18_-DA compositions isolated from the *Delphineae* tribe in the Ranunculaceae family in recent decades. A total of 117 distinct C_18_-DA components, including 58 lappaconitines, 54 ranaconitines, and five rearranged-types, with identified structures have been reported, and these components are mainly from plants from the subgen. *Lycoctonum* in the genus *Aconitum* and less so from the genus *Delphinium*. Natural C_18_-DAs have exhibited a wide range of bioactivities, including analgesic, antiarrhythmic, anti-inflammatory, anti-tumor, and insecticidal activities, which are closely related to their chemical structures. The high chemical diversity among the reported C_18_-DA constituents in *Delphineae* plants indicated their potential as a vast resource for drug discovery. Furthermore, C_18_-DAs in *Delphineae* plants showed chemotaxonomic values and a high regularity of distribution at different taxonomic levels, which could be utilized to serve as good chemical molecular markers in the taxonomic treatment of plants within this tribe especially with infrageneric division.

Although C_18_-DAs in *Delphineae* plants have attracted considerable interest, there is still potential for more research. First, pharmacological investigations on C_18_-DAs are restricted to widespread compounds, especially LA, while most less-common C_18_-DAs are still largely unexplored. The potential of other C_18_-DAs constituents in drug discovery remains ignored, as well as their SAR. More extensive pharmacological studies of other C_18_-DAs are necessary. Furthermore, there are few reported data focused on the toxicity, side effects, and clinical efficiency of C_18_-DAs, which hinders its application and promotion in therapy.

Second, in chemotaxonomic studies, most of the current studies implemented by phytochemists are still aiming at discovering compounds with new structures and are not aiming at illuminating or characterizing the chemical constituent profiles of certain plants. Phytochemists usually prefer to publish new compounds, and known or common C_18_-DAs are largely ignored and are often not reported. Thus, only a few C_18_-DAs have been reported in a certain species, and fewer than five C_18_-DAs have been reported for most of these investigated species. The data on C_18_-DAs in *Delphineae* plants are still insufficient and fragmentary, and more complete reports based on extensive investigations are needed. In addition, in the reported phytochemical studies on C_18_-DAs in *Delphineae*, the content of certain C_18_-DA in plants is lacking, which has also been reported as a key reference for taxonomy that reveals evolutionary degrees. Chemotaxonomic studies on C_18_-DA composition in the *Delphineae* tribe should also consider the content in addition to the chemical structural diversity of metabolites. While little information can be obtained during the conventional process of studying phytochemicals, incorporating conventional analysis methods such as HPLC, UV, or MS to acquire the contents of these important chemical markers is encouraged in further research.

## Conflicts of interest

The authors declare no conflict of interest.

## Supplementary Material

## References

[cit1] Jabbour F., Renner S. S. (2011). Taxon.

[cit2] DuPasquier P. E., Andro-Durand V., Batory L., Wang W., Jabbour F. (2021). PhytoKeys.

[cit3] Jabbour F., Renner S. S. (2012). Mol. Phylogenet. Evol..

[cit4] Wang W. C. and Michael J. W., Flora of China, 2001

[cit5] Wang W. C. (2019). Guihaia.

[cit6] Benn M. H., Okanga I. F., Manavu R. M. (1989). Phytochemistry.

[cit7] Ali S., Chouhan R., Sultan P., Hassan Q. P., Gandhi S. G. (2021). Adv. Tradit. Med..

[cit8] Yin T. P., Cai L., Ding Z. T. (2020). RSC Adv..

[cit9] Yin T. P., Cai L., Ding Z. T. (2020). RSC Adv..

[cit10] Zhou G., Tang L., Zhou X., Wang T., Kou Z., Wang Z. (2015). J. Ethnopharmacol..

[cit11] Nyirimigabo E., Xu Y., Li Y., Wang Y., Agyemang K., Zhang Y. (2015). J. Pharm. Pharmacol..

[cit12] Yin T. P., Yan Y. F., Yang X. Y., Li W. (2021). Biochem. Syst. Ecol..

[cit13] Wang F. P., Chen Q. H., Liu X. Y. (2010). Nat. Prod. Rep..

[cit14] Luo Y. (2005). Acta Phytotaxon. Sin..

[cit15] Ichinohe Y. (1978). J. Japan. Chem..

[cit16] Xiao P. G., Wang F. P., Gao F., Yan L. P., Chen D. L., Liu Y. (2006). J. Syst. Evol..

[cit17] Hao X. J., Yang C. R., Chen S. Y., Zhou J. (1985). J. Syst. Evol..

[cit18] Gao F., Zhu S. A., Wu W., Wang X. G., Song L. (2010). Biochem. Syst. Ecol..

[cit19] Cook D., Manson J. S., Gardner D. R., Welch K. D., Irwin R. E. (2013). Biochem. Syst. Ecol..

[cit20] Shen Y., Liang W. J., Shi Y. N., Kennelly E. J., Zhao D. K. (2020). Nat. Prod. Rep..

[cit21] Yunusov M. S. (1991). Nat. Prod. Rep..

[cit22] Wang F. P. , Chen Q. H. and Liang X. T., The C18-diterpenoid alkaloids, 2009, pp. 1–78 10.1016/s1099-4831(09)06701-7 19827365

[cit23] Rosendahl H. V. (1896). J. Pharm..

[cit24] Schulze H., Ulfert F. (1922). Arch. Pharm..

[cit25] Mollov N., Tada M., Marion L. (1969). Tetrahedron Lett..

[cit26] Tel'nov V. A., Yunusov M. S., Rashkes Y. V., Yunusov S. Y. (1971). Chem. Nat. Comp..

[cit27] Murav'eva D. A., Plekhanova T. I., Yunusov M. S. (1972). Chem. Nat. Comp..

[cit28] Tel'nov V. A., Yunusov M. S., Yunusov S. Y. (1973). Chem. Nat. Comp..

[cit29] Wang F. P., Fang Q. C. (1983). Acta Pharm. Sin..

[cit30] Shi X. W., Lu Q. Q., Zhou J. H., Cui X. A. (2015). Acta Crystallogr. E.

[cit31] Shi X. W., Lu Q. Q., Zhou J. H., Cui X. A. (2015). Acta Crystallogr. E.

[cit32] Zhou J. H., Li Y., Zhang L., Wang D. Q. (2012). Nat. Prod. Res..

[cit33] Zeng Z., Qasem A. M. A., Kociok-Köhn G., Rowan M. G., Blagbrough I. S. (2020). RSC Adv..

[cit34] Zeng Z., Kociok-Köhn G., Woodman T. J., Rowan M. G., Blagbrough I. S. (2021). Eur. J. Org. Chem..

[cit35] Deng H. Y., Chen Q. H., Wang F. P. (2014). Nat. Prod. Commun..

[cit36] Zhang Z. T., Wang L., Chen Q. F., Chen Q. H., Chen D. L., Liu X. Y., Wang F. P. (2013). Tetrahedron.

[cit37] Wang F. P., Chen D. L., Deng H. Y., Chen Q. H., Liu X. Y., Jian X. X. (2014). Tetrahedron.

[cit38] Zhou X. L., Chen Q. H., Wang F. P. (2004). Chem. Pharm. Bull..

[cit39] Sham'yanov I. D., Tashkhodzhaev B., Mukhamatkhanova R. F., Sultankhodzhaev M. N., Levkovich M. G., Abdullaev N. D., Antipin M. Y. (2012). Chem. Nat. Comp..

[cit40] Jiang S. H., Song B. Z., Zhou B. N. (1988). Acta Chim. Sin..

[cit41] Ulubelen A., Mericli A. H., Mericli F., Kolak U., Ilarslan R., Voelter W. (1998). Phytochemistry.

[cit42] Mericli F., Mericli A. H., Seyhan G. V., Bahar M., Desai H. K., Ozcelik H., Ulubelen A. (2002). Pharmazie.

[cit43] Bitis L., Suzgec S., Sozer U., Ozcelik H., Zapp J., Kiemer A. K., Mericli F., Mericli A. H. (2007). Helv. Chim. Acta.

[cit44] Yu D. Q., Das B. C. (1983). Planta Med..

[cit45] Xue W. J., Zhao B., Zhao J. Y., Sagdullaev S. S., Akber Aisa H. (2019). Phytochem. Lett..

[cit46] Shamma M., Chinnasamy P., Miana G. A., Khan A., Bashir M., Salazar M., Beal J. L. (1979). J. Nat. Prod..

[cit47] Wang F. P., Peng C. S., Jian X. X., Chen D. L. (2001). J. Asian Nat. Prod. Res..

[cit48] Xue W. J., Zhao B., Kodirova D. R., Zhao J.
Y., Aisa H. A. (2020). Chem. Nat. Comp..

[cit49] Cai L., Chen D. L., Wang F. P. (2006). Nat. Prod. Commun..

[cit50] Tan J. J., Tan C. H., Ruan B. Q., Jiang S. H., Zhu D. Y. (2006). J. Asian Nat. Prod. Res..

[cit51] Srivastava S. K. (1990). Fitoterapia.

[cit52] Jiang S. H., Wang H. Q., Li Y. M., Lin S. J., Tan J. J., Zhu D. Y. (2007). Chinese Chem. Lett..

[cit53] Chen S. Y., Qiu L. G. (1989). Acta Botanica Yunnanica.

[cit54] Yin T. P., Cai L., Fang H. X., Fang Y. S., Li Z. J., Ding Z. T. (2015). Phytochemistry.

[cit55] Nishanov A. A., Sultankhodzhaev M. N., Yunusov M. S., Kondrat'ev B. G. (1991). Chem. Nat. Comp..

[cit56] Nishanov A. A., Tashkhodzhaev B., Usupova I. M., Sultankhodzhaev M. N. (1992). Chem. Nat. Comp..

[cit57] Feng F., Liu W. Y., Chen Y. S., Ye W. C., Liu J. H., Zhao S. X. (2003). J. Chin. Pharm. Univ..

[cit58] Mu Z. Q., Gao H., Huang Z. Y., Feng X. L., Yao X. S. (2012). Org. Lett..

[cit59] Sultankhodzhaev M. N., Boronova Z. S., Nishanov A. A. (1997). Chem. Nat. Comp..

[cit60] Zhao D. K., Ai H. L., Zi S. H., Zhang L. M., Yang S. C., Guo H. C., Shen Y., Chen Y. P., Chen J. J. (2013). Fitoterapia.

[cit61] Zhang Q., Tan J. J., Chen X. Q., Gao Z. B., Jia Q., Chen K. X., Li Y. M. (2017). Tetrahedron Lett..

[cit62] De la Fuente G., Orribo T., Gavin J. A., Acosta R. D. (1993). Heterocycles.

[cit63] Peng C. S., Chen D. L., Chen Q. H., Wang F. P. (2005). Chinese J. Org. Chem..

[cit64] Jiang G. Y., Qin L. L., Gao F., Huang S., Zhou X. L. (2020). Fitoterapia.

[cit65] Shan L. H., Zhang J. F., Chen L., Wang J. X., Huang S., Zhou X. L. (2015). Nat. Prod. Commun..

[cit66] Song L., Liang X. X., Chen D. L., Jian X. X., Wang F. P. (2007). Chem. Pharm. Bull..

[cit67] Shan L. H., Zhang J. F., Gao F., Huang S., Zhou X. L. (2018). J. Asian Nat. Prod. Res..

[cit68] Wang S., Zhou X. L., Gong X. M., Fan X. Y., Lan M. S. (2016). J. Asian Nat. Prod. Res..

[cit69] Hohmann J., Forgo P., Hajdú Z., Varga E., Máthé I. (2002). J. Nat. Prod..

[cit70] Alva A., Grandez M., Madinaveitia A., De la Fuente G., Gavin J. A. (2004). Helv. Chim. Acta.

[cit71] Jiang Q. P., Sung W. L. (1985). Heterocycles.

[cit72] Almanza G., Bastida J., Codina C., De la Fuente G. (1997). Phytochemistry.

[cit73] Kolak U., Ozturk M., Ozgokce F., Ulubelen A. (2006). Phytochemistry.

[cit74] Usmanova S. K., Bessonova I. A., Abdullaev N. D., Levkovich M. G. (1999). Chem. Nat. Comp..

[cit75] Ablajan N., Zhao B., Zhao J. Y., Wang B. L., Sagdullaev S. S., Aisa H. A. (2021). Phytochemistry.

[cit76] Yin T. P., Shu Y., Mei R. F., Wang J. P., Cai L., Ding Z. T. (2018). Biochem. Syst. Ecol..

[cit77] He Y. Q., Ma Z. Y., Yang Q., Du B. Z., Jing Z. X., Yao B. H., Hamann M. T. (2010). Biochem. Syst. Ecol..

[cit78] Mucher W. (1993). Phyton.

[cit79] Zhang J. F., Chen L., Huang S., Shan L. H., Gao F., Zhou X. L. (2017). J. Nat. Prod..

[cit80] Wan L. X., Zhang J. F., Zhen Y. Q., Zhang L., Li X., Gao F., Zhou X. L. (2021). J. Nat. Prod..

[cit81] Hu Z. X., An Q., Tang H. Y., Chen Z. H., Aisa H. A., Zhang Y., Hao X. J. (2019). Phytochemistry.

[cit82] Shu Y., Yin T. P., Wang J. P., Gan D., Zhang Q. Y., Cai L., Ding Z. T. (2018). Chinese J. Nat. Med..

[cit83] Jiang H., Huang S., Gao F., Zhen Y., Li C., Zhou X. (2019). Nat. Prod. Res..

[cit84] Li Y. H., Chen D. H. (1994). J. Integr. Plant Biol..

[cit85] Fan X., Yang L., Liu Z., Lin L., Li C., Guo S., Wang Z., Wang Z., Sui F. (2019). Phytochemistry.

[cit86] Zhang Z. T., Chen D. L., Chen Q. H., Wang F. P. (2013). Helv. Chim. Acta.

[cit87] Li H. Y., Yan B. C., Wei L. X., Sun H. D., Puno P. T. (2021). Nat. Prod. Bioprospect..

[cit88] Zhang J. F., Li Y., Gao F., Shan L. H., Zhou X. L. (2019). J. Asian Nat. Prod. Res..

[cit89] Zhou X., Obaid Arhema Frejat F., Xu W., Shan L. (2017). Heterocycles.

[cit90] Li Y. F., Zheng Y. M., Yu Y., Gan Y., Gao Z. B. (2019). Acta Pharmacol. Sin..

[cit91] Guo X., Tang X. C. (1989). Acta Pharmacol. Sin..

[cit92] Ono M., Satoh T. (1992). Jpn. J. Pharmacol..

[cit93] Ameri A., Metzmeier P., Peters T. (1996). Br. J. Pharmacol..

[cit94] Ono M., Satoh T. (1991). Jpn. J. Pharmacol..

[cit95] Ou S., Zhao Y. D., Xiao Z., Wen H. Z., Cui J., Ruan H. Z. (2011). Neurochem. Int..

[cit96] Wang J. L., Shen X. L., Chen Q. H., Qi G., Wang W., Wang F. P. (2009). Chem. Pharm. Bull..

[cit97] Sun W. X., Dong T. G., Ding C. M. (2012). Adv. Mater. Res..

[cit98] Sun W., Zhang S., Wang H., Wang Y. (2015). Med. Chem. Res..

[cit99] Dzhakhangirov F. N., Sultankhodzhaev M. N., Tashkhodzhaev B., Salimov B. T. (1997). Chem. Nat. Comp..

[cit100] Heubach J. F., Schüle A. (1998). Planta Med..

[cit101] Wright S. N. (2001). Mol. Pharmacol..

[cit102] Vakhitova Iu V., Farafontova E. I., Khisamutdinova R., Iunusov V. M., Cypasheva I. P., Iunusov M. S. (2013). Bioorg. Khim..

[cit103] Yunusov M. S. (2011). Russ. Chem. Bull..

[cit104] Akhiyarov A. A., Lobov A. N., Ivanov S. P., Spirikhin L. V., Gabbasov T. M., Tsyrlina E. M., Yunusov M. S. (2020). Russ. Chem. Bull..

[cit105] Yin T. P., Hu X. F., Mei R. F., Shu Y., Gan D., Cai L., Ding Z. T. (2018). Phytochem. Lett..

[cit106] Zhang J., Li Y. Z., Cui Y. W., Jia P., Yue Z. G., Song B., Song X. M. (2018). Rec. Nat. Prod..

[cit107] Chen L., Zhou X., Qin L., Xing F. (2021). Heterocycles.

[cit108] Liu M. P., Ju Y., Dang Y. L. (2004). Pharmacol. Clin. Chinese Mate. Med..

[cit109] Hu J. M., Xiao L. Y., Pan J. Q., Li J. (2009). J. Chinese Med. Mater..

[cit110] Li Y., Zeng J., Tian Y. H., Hou Y., Da H., Fang J., Gao K. (2021). Phytochemistry.

[cit111] Lin N., Xiao L. Y., Lin P. Y., Zhang D., Chen Q. W. (2005). TCM Res..

[cit112] Hu J. M., Chen F. Y. (2008). China Pharm..

[cit113] Sheng L. H., Xu M., Xu L. Q., Xiong F. (2014). J. Chin. Med. Mater..

[cit114] Ma J. Y., Chen X. l., Hou C. J., Zhu J. Z., Han X. F., Zhang J., Guo H. Y. (2017). Chin. Pharm. J..

[cit115] Zhang X., Ma J., Song N., Guo Y., Hui L., Sang C. (2020). Pharmacology.

[cit116] Qu D. N., Zhang X. M., Sang C. Y., Zhou Y. Q., Ma J. Y., Hui L. (2019). Med. Chem. Res..

[cit117] Qu D., Ma J., Song N., Hui L., Yang L., Guo Y., Sang C. (2020). Acta Histochem..

[cit118] Hui L., Ma J., Song N., Zhang X., Qu D., Sang C., Li H. (2021). Pharmacogn. Mag..

[cit119] Song N., Ma J., Hu W., Guo Y., Hui L., Aamer M., Ma J. (2021). Acta Histochem..

[cit120] Ulubelen A., Mericli A. H., Mericli F., Kilincer N., Ferizli A. G., Emekci M., Pelletier S. W. (2001). Phytother. Res..

[cit121] Reina M., González-Coloma A. (2007). Phytochem. Rev..

[cit122] Chen L., Wang Q., Huang S., Shan L. H., Gao F., Zhou X. L. (2017). Chinese J. Org. Chem..

